# When parks work: Effect of anthropogenic disturbance on occupancy of tropical forest mammals

**DOI:** 10.1002/ece3.6048

**Published:** 2020-04-16

**Authors:** Valentina Oberosler, Simone Tenan, Elise F. Zipkin, Francesco Rovero

**Affiliations:** ^1^ Tropical Biodiversity Section MUSE – Museo delle Scienze Trento Italy; ^2^ Department of Earth and Environmental Sciences University of Pavia Pavia Italy; ^3^ Vertebrate Zoology Section MUSE – Museo delle Scienze Trento Italy; ^4^ Department of Integrative Biology and Ecology Evolutionary Biology and Behavior Program Michigan State University East Lansing Michigan; ^5^ Department of Biology University of Florence Sesto Fiorentino Italy

**Keywords:** bushmeat hunting, camera trapping, hierarchical modeling, snares, Tanzania, Udzungwa

## Abstract

Protected areas (PAs) in the tropics are vulnerable to human encroachment, and, despite formal protection, they do not fully mitigate anthropogenic threats to habitats and biodiversity. However, attempts to quantify the effectiveness of PAs and to understand the status and changes of wildlife populations in relation to protection efficiency remain limited. Here, we used camera‐trapping data collected over 8 consecutive years (2009–2016) to investigate the yearly occurrences of medium‐to‐large mammals within the Udzungwa Mountains National Park (Tanzania), an area of outstanding importance for biological endemism and conservation. Specifically, we evaluated the effects of habitat and proxies of human disturbance, namely illegal hunting with snares and firewood collection (a practice that was banned in 2011 in the park), on species' occurrence probabilities. Our results showed variability in species' responses to disturbance: The only species that showed a negative effect of the number of snares found on occurrence probability was the Harvey's duiker, a relatively widespread forest antelope. Similarly, we found a moderate positive effect of the firewood collection ban on only the suni, another common antelope, and a negative effect on a large opportunistic rodent, the giant‐pouched rat. Importantly, we found evidence of temporal stability in occurrence probability for all species over the 8‐year study period. Our findings suggest that well‐managed PAs can sustain mammal populations in tropical forests. However, variability among species in their responses to anthropogenic disturbance necessitates consideration in the design of conservation action plans for multiple taxa.

## Graphical abstract

Protected areas (PAs) in the tropics are vulnerable to human encroachment, but attempts to quantify their effectiveness remain limited. We used camera‐trapping data collected over 8 consecutive years to evaluate the effects of proxies of human disturbance on the yearly occurrences of forest mammals within the Udzungwa Mountains National Park (Tanzania). Our results showed variability in species' responses to disturbance, and we also found evidence of temporal stability in occurrence probability for all species over the study period, suggesting that well‐managed PAs can sustain mammal populations in tropical forests.

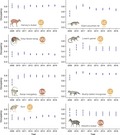

## 1 INTRODUCTION

Tropical forests are the most biologically rich ecosystems on earth (Myers, Mittermeier, Mittermeier, Fonseca, & Kent, 2000), and their rapid disruption imperils global biodiversity more than any other contemporary phenomenon (Gibson et al., 2011). With deforestation advancing quickly, protected areas (PAs) are becoming critical refuges for threatened species, and growing concern about the impacts of anthropogenic activities on tropical biodiversity has led to increases in their number and extent (Jenkins & Joppa, 2009). However, many PAs in the tropics are vulnerable to human encroachment (Bruner, Gullison, Rice, & Fonseca, 2001; Laurance et al., 2012) and do not fully mitigate threats to habitat and biodiversity. This is due to multiple reasons including chronic understaffing, inadequate funding, and political instability hampering adequate law enforcement (Bruner et al., 2001; Laurance et al., 2012; Naughton‐Treves, Holland, & Brandon, 2005; Tranquilli et al., 2014). For these reasons, PAs are not always effective for preventing wildlife declines, and local extirpations have increased (Craigie et al., 2010). Yet, efforts to quantify the effectiveness of PAs and understand whether they can sustain biodiversity remain limited (Geldmann et al., 2013; Leverington, Hockings, & Costa, 2008), and are often based on secondary/qualitative data (Beaudrot et al., 2016; Struhsaker, Struhsaker, & Siex, 2005).

Here, we used camera‐trapping data collected over 8 consecutive years (2009–2016) to investigate the temporal occurrences of mammals within the Udzungwa Mountains National Park (UMNP, Tanzania), an area of outstanding importance for biological endemism and biodiversity conservation (Rovero, Menegon, et al., 2014). Thanks to efficient law enforcement, this park is relatively well protected from extractive uses, especially if related to other PAs across the Udzungwa Mountains (Oberosler, Tenan, Zipkin, & Rovero, 2019 and see Discussion). Yet, instances of human disturbance, specifically hunting and logging, have been regularly reported (Rovero, Mtui, Kitegile, & Nielsen, 2012). Our main objective was to investigate the effect of habitat variables and different sources of human disturbance on occurrence probabilities of target forest mammal species over the 8‐year period. Wire snares are the most frequently used hunting method in African forests because they are inexpensive, effective, and easy to obtain, set, and conceal (Noss, 1998). Snaring is difficult to control by management authorities (Jones, Hawes, Norton, & Hawkins, 2019), and being nonselective, it confers by‐catch mortality on a variety of species (Lindsey et al., 2011). Another potential source of disturbance in the UMNP was firewood collection by adjacent villagers, permitted until 2011. Firewood collection is widespread and increasing across Africa. In this region, fuelwood is the primary energy supply adopted by approximately 70% of the local population (Bailis, Ezzati, & Kammen, 2005). Selective firewood harvesting is a source of forest degradation, especially in fragmented and densely populated landscapes, when biomass removal exceeds forest productivity (Specht, Pinto, Albuquerque, Tabarelli, & Melo, 2015). Furthermore, it may change forest composition and ecosystem functioning, with cascading effects on the structure of wildlife populations and communities (Naughton‐Treves, Kammen, & Chapman, 2007). For example, the harvesting of trees from old‐growth African forest may result in slow‐growing species being replaced with faster growing secondary species, which do not provide food for frugivorous birds or primates (Struhsaker, 1997). Such patterns of arrested forest succession and biotic homogenization have already been documented in South America, where woodfuel harvesting has been shown to be also correlated with hunting (Specht et al., 2015).

We focused on terrestrial, ground‐dwelling, medium‐to‐large mammals that are relatively easily detected by camera traps and particularly sensitive to ground‐occurring human disturbance (e.g., Ahumada et al., 2011). Mammals are key components of tropical ecosystems as they perform critical functions such as predation (Sinclair, Mduma, & Brashares, 2003; Terborgh et al., 2001), grazing (Pringle, Young, Rubenstein, & McCauley, 2007), and seed dispersal (Fragoso, Silvius, & Correa, 2003; Hoffmann et al., 2011). Furthermore, mammals include many charismatic species that are important flagships for conservation (Leader‐Williams & Dublin, 2000; Rondinini, Rodrigues, & Boitani, 2011). We used occupancy (i.e., the estimated probability of a species occurrence at a collection of sites; MacKenzie et al., 2002) as the metric of choice to address our objectives. Given its documented correlation with abundance (MacKenzie, Nichols, Hines, Knutson, & Franklin, 2003), occupancy provides information on both density and extinction likelihood (MacKenzie, Nichols, Sutton, Kawanishi, & Bailey, 2005). Furthermore, this metric accounts for imperfect detection (MacKenzie, 2005) and thus can adequately address the problem that species may go undetected where present. Issues of imperfect detection are ubiquitous in ecological field data and if unaddressed can bias inferences on habitat relationships and “true” species occurrence (Kéry, 2011). We expected a positive effect of the firewood collection ban on species‐specific occurrence probabilities and a negative effect of the number of snares on the relatively few species that may be more vulnerable to such disturbances. However, we also expected average occurrence probability for the majority of species to be relatively stable over the 8‐year period, due to the documented effectiveness of management in the park (Oberosler et al., 2019).

## 2 METHODS

### 2.1 Study area

**Figure 1:**
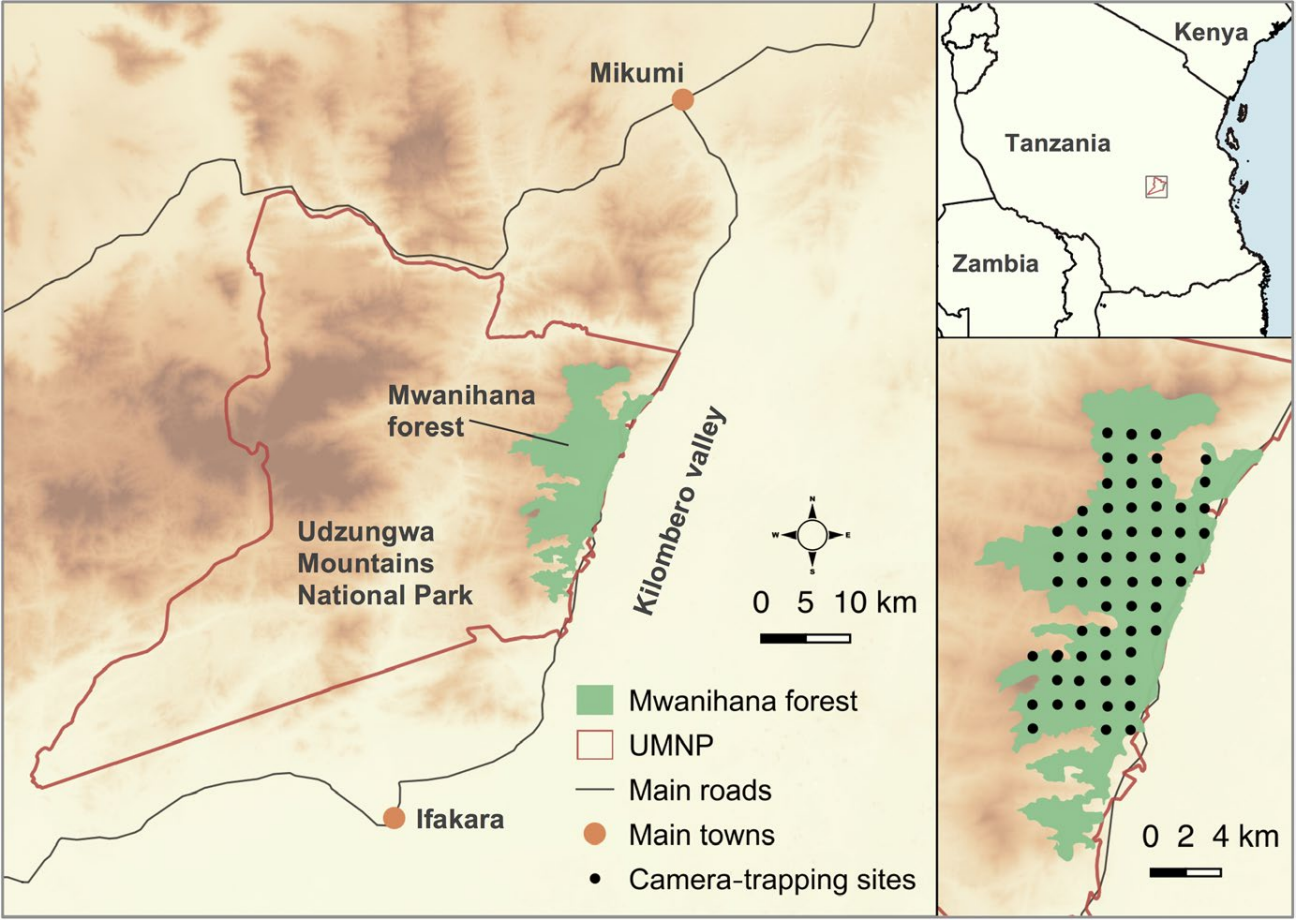
Map of the study area, Mwanihana Forest, in the Udzungwa Mountains of Tanzania. This forest represents the interface between the densely settled Kilombero Valley and the Udzungwa Mountains National Park (UMNP). Camera trap sites are shown as black dots. The geographic location of the study area in Tanzania is shown in the map on the upper right

We conducted our study in Mwanihana Forest (MW, centered on 36°46′E, 7°47′S), located in the UMNP, which was established in 1992 (Figure 1). The UMNP (1,990 km^2^) contains large areas of mountain forest and grassland and is the only national park that supports significant Eastern Arc habitat in Tanzania (Burgess et al., 2007). It is managed by the Tanzania National Parks Authority (TANAPA), a well‐resourced national agency (Rovero, Martin, Rosa, Ahumada, & Spitale, 2014), with UMNP having a mean annual budget allocated for forest management of approximately 400,000 USD, while the number of permanent staff units amounts to more than 70 people (Hegerl et al., 2015). There was no significant variation in patrolling effort during the study period. MW is an east‐facing escarpment slope, characterized by a unique continuous vegetation cover from lowland (300–800 m a.s.l.), submontane deciduous forest (800–1,400 m) to evergreen moist montane forest (>1,400 m; Lovett, 1993). The forest extends over 177 km^2^, with elevation ranging between 290 and 2,300 m a. s. l. The anthropogenic pressure in MW comes mainly from villages located along the eastern side of the forest, where the fertile land provides for commercial production of sugar cane and rice (Hegerl et al., 2015). Agriculture is the most important livelihood activity for local people in the area (Harrison, 2006). MW and the forests nearby act as an important water catchment, securing irrigation and drinking water to many thousands of people (Rovero et al., 2012). Earlier assessments showed that active law enforcement in MW resulted in virtually no disturbance (DeFries et al., 2010), even though evidence of illegal hunting, mainly from snares set to catch ungulates, has been repeatedly recorded, evenly across the forest and especially in the interior zones (Oberosler et al., 2019; Rovero et al., 2012; this study). Another potential source of disturbance is firewood collection by adjacent villagers; this practice was allowed weekly only within 1 km off the PA boundary, but was banned in June 2011.

### 2.2 Target species

We conducted our analysis on eight ground‐dwelling forest species that were recorded every year between 2009 and 2016 in MW with sufficient independent events to ensure identifiability of model parameters (see Appendix 1). These species are characterized by different conservation statuses, trophic positions, and relative abundance, representing the variability within the larger mammal community. We included the (a) Harvey's duiker (*Cephalophus harveyi*), a medium‐bodied forest antelope (average body mass 14.50 kg) that is common but declining across its range, and the (b) suni (*Nesotragus moschatus*), which is a small antelope (6.50 kg) that remains common through parts of its range but locally threatened. Both these forest antelopes show preference for low‐to‐medium, dense vegetation and are thus considered lowland forest dwellers (Rovero, Martin, et al., 2014). The IUCN‐vulnerable (c) gray‐faced sengi (*Rhynchocyon udzungwensis*) is a recently discovered insectivore (0.75 kg), endemic of two forests of the Udzungwa Mountains (IUCN, 2016; Rovero, Martin, et al., 2014) and typically occurring at high elevations. Two other species included in the analyses and listed as IUCN‐endangered (IUCN, 2016) are the Udzungwa's endemic and iconic monkey (d) Sanje mangabey (*Cercocebus sanjei*; 8.00 kg), and the poorly known large‐bodied (e) Abbott's duiker (*Cephalophus spadix*; 56.00 kg), endemic of highland forests of Tanzania. Both of these species occur more frequently at high elevations, showing preference for the interior montane forest (Rovero, Martin, et al., 2014). The widespread (f) giant‐pouched rat (*Cricetomys gambianus*) is a strictly nocturnal large omnivore (1.30 kg). Targeted mesocarnivores included the (g) bushy‐tailed mongoose (*Bdeogale crassicauda*), widely distributed in eastern Africa and often sighted near the forest edge (1.50 kg), and the (h) Lowe's servaline genet (*Genetta servalina lowei*), a poorly known subspecies endemic of the Eastern Arc Mountains (1.06 kg; body mass data from Smith et al., 2003).

### 2.3 Data collection

Camera‐trapping data were collected yearly during 2009–2016 in MW as part of the Tropical Ecology Assessment and Monitoring (TEAM) Network program (Rovero & Ahumada, 2017). Every dry season (July–November), Reconyx RC45 and HC500 (Reconyx Inc.) camera traps were distributed over three consecutive arrays of 20 cameras according to a standardized protocol for monitoring terrestrial vertebrates (TEAM Network, 2011), for a total of 60 sampling sites (Figure 1). Cameras were placed at a density of one camera per 2 km^2^, and locations were selected to be representative of the habitat and elevation gradient of the forest, resulting in sampling sites covering the whole forest extent and elevation range (see TEAM Network, 2011 for details about the protocol). Every sampling season, each camera was placed on a tree to record a trail segment approximately 2–3 m away and deployed for a minimum of 30 days (generally 30–35 days). The area within the sensor field of the camera was cleared of ground vegetation for better visibility. Functioning camera traps (58–60 per year, mean = 59.3) accumulated 14,743 camera days (31.1 mean per camera, 1842.9 mean per year). Sampling yielded 141,541 images of 31 wild mammal species (see Appendix 1 for detection events per year).

### 2.4 Data analyses

**Figure 2:**
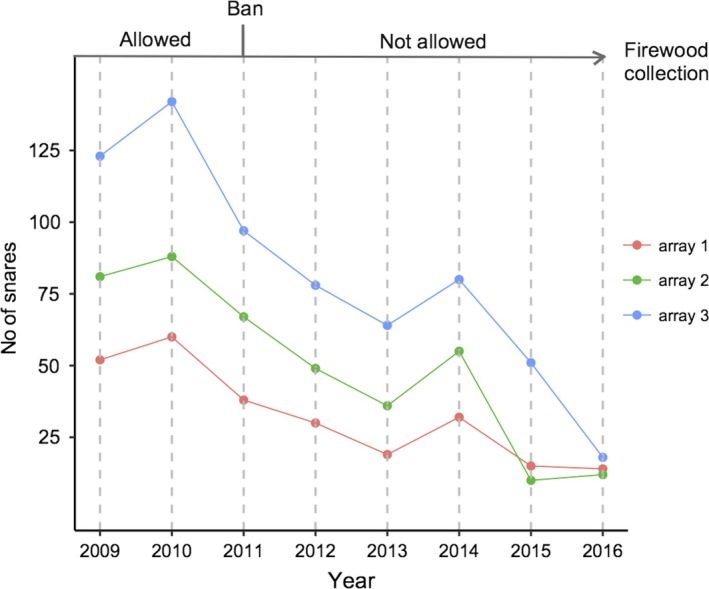
Summary of the year‐dependent covariates used in the species‐specific occupancy models of eight mammal species in the Udzungwa Mountains of Tanzania. At the top, the management status of the forest in terms of firewood collection (used as binary 0–1 year‐specific covariate). Below, the number of snares for illegal hunting collected by the field team during the deployment of each array of camera traps across years

We summarized species‐specific detection/nondetection data at each site *i*, sampling occasion *j*, during year *k*, in 3‐d arrays *Y_i,j,k_*, using a sampling occasion of 5 days. Such resolution was small enough to yield stable parameter estimates and large enough to ensure the model was computationally tractable. We carried out single‐species occupancy analyses using a hierarchical modeling framework (MacKenzie et al., 2002). The observational data, *y_i,j,k_*, denoted detections (*y* = 1) or nondetection (*y* = 0) for each species at site *i*, sampling occasion *j*, during year *k*. True occurrence was only partially observable and was modeled as a Bernoulli random variable, z*_i,k_* ~ Bern(*ψ_i,k_*) with probability *ψ_i,k_*, where *z* = 1 when the species was present at site *i* during year *k*, and zero otherwise. We modeled occurrence probability as a function of elevation (m a.s.l., denoted ELEV), as recorded at each camera site by using handheld GPS units, which we considered a proxy for variation in forest composition over the study area and hence habitat type (Lovett, 1993; Lovett, Marshall, & Carr, 2006). We also included a square term for the effects of elevation, to account for potential quadratic relationships between altitude and species‐specific distribution (ELEV2). The number of snares (SNARES) for illegal hunting collected by the field team during the deployment of each array of camera traps in every sampling season was used as a yearly‐site continuous covariate and a proxy for the amount of illegal hunting at sites. We also included a binary (0–1) year‐specific covariate to describe the management status of the forest regarding the firewood collection activity (BAN), to test for potential effects of its ban on species‐specific occurrence probabilities (BAN = 0 for 2009–2011 and BAN = 1 for 2012–2016, respectively; Figure 2 and Table 1). We developed an autologistic model, such that the intercept term in the occurrence model was dependent on whether or not the species was present at site *j* in the previous year (Zipkin, Grant, & Fagan, 2012). Compared to the full colonization/extinction model, this is considered to be a more convenient parameterization when covariates are thought to influence occupancy, rather than its components colonization and persistence (Royle & Dorazio, 2008). Furthermore, in such cases the autologistic formulation appears to yield a more efficient Bayesian implementation. We thus assumed that the *logit* transformation of the occurrence probability is a linear combination of the autologistic component and the effects of covariates as follows:

$$\text{logit}\left(\psi_{i,k}\right)=\alpha 0+\alpha 1*z_{i,k-1}+\alpha_{S}*{\text{SNARES}}_{i,k}+\alpha_{E}*{\text{ELEV}}_{i}+\alpha_{E2}*\text{ELEV}2_{i}+\alpha_{B}*{\text{BAN}}_{k}$$

Here, (*α*0* + α*1*z_i,k__−1_) is the intercept term, where inverse‐logit(*α*0) is the species' colonization probability and inverse‐logit(*α*0* + α*1) the persistence probability, while *α*_S_, *α*_E_, *α*_E2_, and *α*_B _are the effect of covariates on occupancy. The linear predictor of occurrence probability in the first year was similarly formulated as follows:

$$\text{logit}\left(\psi_{i,1}\right)=\alpha 2+\alpha_{S}*{\text{SNARES}}_{i,1}+\alpha_{E}*{\text{ELEV}}_{i}+\alpha_{E2}*\text{ELEV}2_{i}$$

**Table 1:** Environmental and disturbance covariates fitted in dynamic occupancy models for mammals detected over a period of 8 years in the Udzungwa Mountains of Tanzania

Variable	Notation	Description	Hypotheses
Elevation	ELEV	Elevation of camera‐trapping sites (m a.s.l.).	Species‐specific variability in response to elevation as proxy for habitat type, for example, positive effect on interior species (Sanje mangabey, Abbott's duiker).
Elevation squared	ELEV2	Square term for the effect of elevation.	Mid‐altitude peaks in species‐specific distribution for species ranging across the elevation gradient (e.g., suni).
*N* of snares	SNARES	The number of snares for illegal hunting collected by the field team during the deployment of each array of camera traps in every sampling season.	Negative effect especially for species most targeted by hunting, for example, forest antelopes.
Firewood collection activity	BAN	Binary year‐specific covariate, which describes the state of the forest in terms of firewood collection activity (allowed/not allowed). Firewood collection was allowed in the forest until 2011.	Positive effect for species preferring edge/low elevation species (e.g., Harvey's duiker, suni)

Note

The hypothesized relationship is also indicated.

We did not include the SNARE covariate in the occupancy model for the gray‐faced sengi because, given its small size, this species is not affected by hunting with snares (F. Rovero, unpublished data). Indeed, it was the smallest species among the analyzed ones (see Target Species).

Occurrence is imperfectly observed, which confounds the estimation of *ψ_i,k_*. We therefore specified the detection model for the observational data as *y_i,j,k_* ~ Bern(p_i_*z_i,k_), a Bernoulli random variable dependent on the occupancy state, where *p* is the detection probability at site *i,* given that the species is present. We expected detection *p_i_* to vary based on the shortest linear distance to the park border (BORDER), a continuous site covariate. We hypothesized that animals would be more elusive near the forest edge because of greater disturbance and, possibly, denser forest floor vegetation cover due to lowered canopy height and increased light penetration through the understory, both limiting detection by camera traps (Baez & Balslev, 2007; Harper et al., 2005; Laurance et al., 2002; Rovero, Menegon, et al., 2014). The detection model for each species at site *i* was specified as follows:

$$\text{logit}\left(p_{i}\right)=\beta 0+\beta_{B}*{\text{BORDER}}_{i}$$

We fit the model using a Bayesian formulation and Markov chain Monte Carlo using JAGS (Plummer, 2003), called from R (R Development Core Team, 2016) through the package “jagsUI” (Kellner, 2016). For each model, we ran three chains of length 100,000, discarded the first 5,000 iterations as burn‐in, and thinned the remaining results by taking each 20th value from the chains. Continuous covariates were derived using geoprocessing tools available in QGIS 2.18.0 (QGIS Development Team, 2017) and standardized to have mean zero and unit variance. Elevation and distance from border were collinear (Pearson's *r* = .7), but this did not affect the analysis since their effects were included on different parts of the model. Finally, we did not include a year effect on occupancy probability because it was inversely correlated with snares (Pearson's *r* = .7; see Figure 2). We used uninformative priors (see Appendix S1) and verified convergence through visual inspection of the chains and with the Gelman–Rubin diagnostic (Brooks & Gelman, 1998). Finally, we evaluated the goodness of fit of the models. We assessed the adequacy of the models using the discrepancy measure and approach suggested by Gelman, Meng, and Stern (1996), that is, by computing the Bayesian *p*‐value (see also Zipkin, DeWan, & Royle, 2009). Extreme values (e.g., <.05 or >.95) indicate that the model is inadequate.

## 3 RESULTS

**Figure 3:**
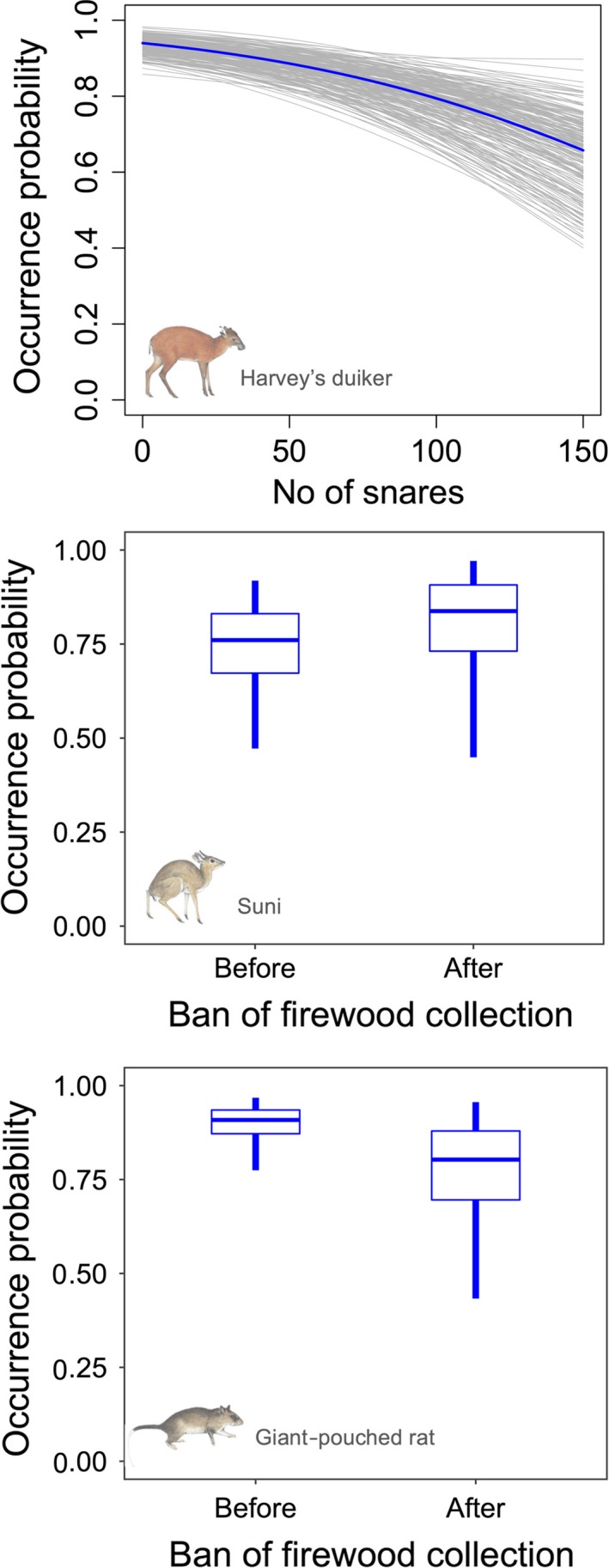
Significant effects of disturbance covariates on occurrence probability for eight mammal species detected with camera traps in the Udzungwa Mountains, Tanzania. Drawings by J. Kingdon reproduced with permission

**Figure 4:**
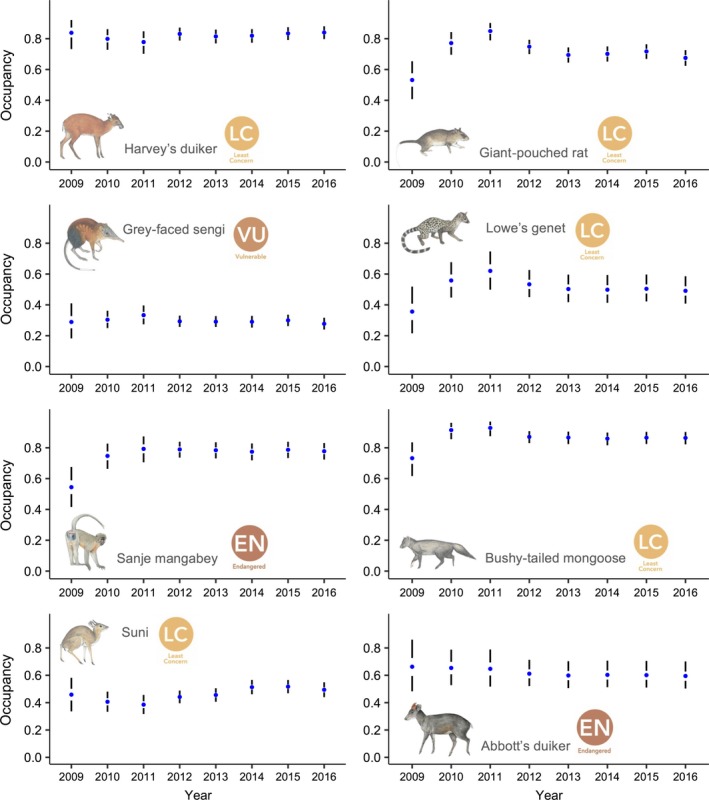
Estimated mean occupancy in the 8‐year period for eight mammal species camera‐trapped in the Udzungwa Mountains, Tanzania. Drawings by J. Kingdon reproduced with permission

The effects of covariates on occupancy varied among species (Figure 3, Appendices 2 and 3). The only species for which the number of snares collected had a significant effect on occupancy was the Harvey's duiker, showing a negative effect (*α*_S_: mean −0.49, 95% Bayesian credible interval [BCI] −0.76 to −0.21). Firewood collection ban had a positive, but marginally nonsignificant effect (i.e., BCI overlapped 0) on the occurrence probability of the suni (*α*_B_: mean 0.49, 95% BCI −0.09 to 1.09). On the contrary, the ban had a negative effect on the giant‐pouched rat (*α*_B_: mean −0.90, 95% BCI −1.50 to −0.32; Figure 3 and Appendix 2). Model results also showed that occurrence at a site in one year had a strong influence on species occurrence probabilities in the following year, revealing temporal consistency in occupancy for most species across sites (Appendix 4). Specifically, persistence (*α*0* + α*1) was significantly higher than colonization (α*0*) for all species, except for the bushy‐tailed mongoose and the Abbott's duiker (Table 2). Furthermore, the derived species‐specific average occurrence probability showed stability for all species (Figure 4). Elevation (*α*_E_ + *α*_E2_) had variable effects on species‐specific occurrence probabilities, which were significant and strong for most species (see Appendices 2 and 3). Contrary to patterns in occupancy, detectability varied more consistently among species and there was an effect of distance to park border for all species except the Sanje mangabey and suni (see Appendix 2). Bayesian *p*‐values computed for each model were all in the range .44 < *p*<.49, suggesting that the models for all species provided an adequate fit to the data.

**Table 2:** Parameter estimates from autologistic models of occurrence probability of eight species of mammals monitored by camera trapping in the Udzungwa Mountains of Tanzania

Species	Colonization	Persistence
Harvey's duiker	0.71 (0.55–0.84)	0.88 (0.80–0.93)
Gray‐faced sengi	0.08 (0.04–0.17)	0.84 (0.68–0.93)*
Sanje mangabey	0.75 (0.60–0.87)	0.93 (0.85–0.97)
Suni	0.19 (0.11–0.30)	0.76 (0.63–0.86)*
Bushy‐tailed mongoose	0.89 (0.76–0.96)	0.95 (0.90–0.98)
Abbott's duiker	0.60 (0.35–0.84)	0.78 (0.59–0.92)
Lowe's genet	0.44 (0.30–0.59)	0.75 (0.59–0.87)*
Giant‐pouched rat	0.59 (0.45–0.72)	0.91 (0.84–0.95)*

Note

Values are means with 95% Bayesian credible intervals (BCI) of colonization (*α*0) and persistence (*α*0* + α*1) probabilities for each species. Asterisks denote no overlap between 95% BCIs.

## 4 DISCUSSION

Using systematic camera‐trapping data and hierarchical modeling, we studied the occurrences of forest mammals over 8 consecutive years (2009–2016) within the Udzungwa Mountains National Park (Tanzania). We found that target species' occurrence probability over the study period was stable in spite of illegal hunting, and firewood collection until 2011, by adjacent villagers. The responses of the target species to these sources of disturbance were variable. The Harvey's duiker was the only species negatively affected by snares. Although wire snares can potentially catch a pool of similar size species, this common antelope may be particularly vulnerable, and hunters probably place snares along trails that duikers routinely use. In addition, the Harvey's duiker is among the most abundant species in our dataset, hence the effect of illegal hunting may be greatest. Notably, we observed a forest‐wide decline in poaching over the 8‐year period as the number of snares collected across the study area decreased (Figure 2). Since the number of snares was inversely correlated with the sampling year, the result for the Harvey's duiker may mirror an increase in occurrence probability for this species. Furthermore, although this metric may not measure accurately the actual poaching pressure across the forest, given that hunters may change their target areas periodically, this reduction, coupled with firewood collection ban in 2011, suggests a gradual decrease of human pressure in the PA and/or an improvement of management effectiveness. Firewood collection was permitted only within the lower elevation portion of the forest near the park boundary (Hegerl et al., 2015), hence of potentially negligible impact on species of interior forest. We expected a positive effect of the ban on occupancy trends mainly for edge‐dwelling species (such as the Harvey's duiker and the suni). Interestingly, results showed a small positive effect of the ban only for the suni, suggesting that the impact of this practice on other species may have been minor. This result, however, could also be attributed to the limited amount of data available before the ban. Moreover, we found an opposite effect on occupancy for a relatively common species, the giant‐pouched rat. This opportunistic species is reported to fare well in highly disturbed habitats, and thus, the result is not surprising (Engeman et al., 2006; Hegerl et al., 2015; Roemer, Gompper, & Van Valkenburgh, 2009).

Our findings suggest that law enforcement in the target PA is effective in sustaining the target populations, and support evidence from previous studies that the national park is more effective as compared to other reserves in the area where ground protection appears inadequate (Jones et al., 2019; Rovero et al., 2012). This is also consistent with results from a previous study over the same study area (Rovero et al., 2015), which reported that estimated group abundance of arboreal primate species appeared stable over an 11‐year period (2002–2012). In contrast, most species declined severely in a nearby nature reserve, due to high levels of uncontrolled illegal hunting (Hegerl et al., 2015; Oberosler et al., 2019; Rovero et al., 2015). These authors report a marked difference between this nature reserve and MW in terms of PA management indicators, that is, the annual budget allocated for forest management (USD c. 1,000 vs. 400,000, respectively) and the number of permanent staff units (1 vs. 78, respectively, with the latter number, related to the whole national park, including 60 rangers that are regularly involved in patrols). Our species‐specific results on population trends are also consistent with the community‐level stability detected by Beaudrot et al. (2016) in a previous study using a shorter time series of data from across the TEAM network. Thus, our findings are generally in line with global assessments suggesting less extreme deterioration in tropical forest protected areas than reports based on data lacking of standardized monitoring infrastructures (Beaudrot et al., 2016; Laurance et al., 2012).

The forest antelope populations we monitored over 8 years across Mwanihana Forest seem to be in stable numbers. These species in the middle level of the trophic web play a vital ecological role in the stability of forest ecosystems, as both seed dispersers and prey (Wilson, 2001). Tropical ungulates are the most heavily hunted species across African tropical moist forests (Abernethy, Coad, Taylor, Lee, & Maisels, 2013; Fa & Brown, 2009; Lwanga, 2006). That forest antelopes in the UMNP seem stable and relatively widespread as compared to other African parks is confirmed by another recent study (O'Brien et al., 2019). Significant declines have been previously documented for *Philantomba monticola* and *Cephalophus spadix* across some forest reserves in Udzungwa (Nielsen, 2006), whose protection has been recorded as less effective (Jones et al., 2019; Oberosler et al., 2019). Yet, small levels of illegal hunting and human disturbance seem to have no direct effect on duikers' occupancy patterns over time in the target national park. This is likely a general trend across African National Parks, where most of the duikers' declines are not directly associated with hunting (O'Brien et al., 2019).

Elevation had a significant effect on occupancy for five of the eight species examined. We considered elevation a proxy for preference of moist evergreen forest interior (which occurs at higher elevation) and it likely also reflects decreasing human disturbance, with human settlements bordering the eastern edge of the low elevation forest. Results showed a strong and positive effect of elevation on occurrence probability for the gray‐faced sengi. This matches earlier findings (Rovero, Martin, et al., 2014). We also found no significant effect of the ban of firewood collection on occupancy for the gray‐faced sengi, suggesting that human disturbance may only have a minor impact on this species over the study area. This recently discovered insectivore, assessed as vulnerable by the IUCN (IUCN, 2016), is endemic of only two forests across the Udzungwa Mountains (Rovero et al., 2008), and our results are encouraging about its conservation. Results for the Harvey's duiker and the bushy‐tailed mongoose showed a negative effect of elevation on occupancy. Results for the suni antelope showed a mid‐altitude peak in its distribution with the highest occupancy probability estimates below 1,000 m a. s. l. and so close to human disturbance sources, suggesting a higher sensitivity of this species to anthropogenic disturbance and higher potential risk of extinction than other species of the interior forest and high elevations. This is consistent with the above‐mentioned positive effect of the firewood collection ban on occupancy showed for this species.

Given the high investment that has been made worldwide in PAs (Balmford, Gaston, Blyth, James, & Kapos, 2003) and their importance for long‐term conservation, it is crucial to document how effectively they are performing (Naughton‐Treves et al., 2005). In this context, a key information challenge in conservation practice is the quality of available biodiversity data in the tropics (Beaudrot et al., 2016). In particular, there is a lack of primary in situ data on populations in tropical PAs, which results in conclusions based on aggregated secondary data and expert opinion (Geldmann et al., 2013). In this regard, our study highlights the effectiveness of systematic camera trapping coupled with hierarchical models to investigate the status of mammal communities and to assess species‐specific responses to anthropogenic variables (Pettorelli, Lobora, Msuha, Foley, & Durant, 2010; Zipkin, Royle, Dawson, & Bates, 2010). This approach is particularly effective when applied to standardized and long‐term data series, which are needed for long‐lived animals. In conclusion, our findings provide evidence that effective ground protection is associated with stability over time in the occurrence probability of a pool of commonly camera‐trapped species of forest mammals, and therefore, we support the notion that legal protection backed up by on‐ground protection can maintain diverse mammal communities in tropical forests. Yet, with ever‐growing human population density in Tanzania, and the current paving of the road that runs east of the park boundary, illegal encroachment is likely to increase in the near future. Hence, we recommend patrolling efforts be focused in the areas close to the forest edge, and also in regions with the highest number of snares for illegal hunting (array 3). Conservation measures should include the creation of buffer zones to decrease human pressure on the forest edge (e.g., Cavada, Havmøller, Scharff, & Rovero, 2019) and a continued monitoring of the status of wildlife populations. Finally, integrated development and conservation projects should concentrate their activities in villages adjacent to the forest, to raise awareness about sustainable forest use and the importance of preserving its wildlife, while engaging locals in monitoring and patrolling initiatives. Together with long‐term management effectiveness, the support of communities to establish livelihood alternatives to bushmeat hunting may be a way to reduce illegal hunting across the UMNP.

## CONFLICT OF INTERESTS

The authors declare that they have no conflict of interest.

## AUTHOR CONTRIBUTIONS

FR designed the study, ensured funding, and contributed to fieldwork; VO analyzed the data together with ST and EZ; VO led the writing of the manuscript to which FR, ST, and EZ contributed. All authors gave final approval for publication.

## Data Availability

The dataset generated and analyzed during the current study is available in the figshare repository (https://doi.org/10.6084/m9.figshare.11566764).

## APPENDIX 1

Species detected by camera traps during 2009–2016 in Mwanihana Forest (Udzungwa Mountains National Park, Tanzania) with the number of independent detection events (i.e., separated by 1 day). In bold are the eight species targeted by the analyses

**Table:**

		2009	2010	2011	2012	2013	2014	2015	2016
1	*Atilax paludinosus*	3	3	12	5	6	4	1	6
2	***Bdeogale crassicauda***	**126**	**267**	**259**	**259**	**336**	**304**	**289**	**341**
3	***Cephalophus harveyi***	**281**	**197**	**228**	**260**	**290**	**344**	**293**	**356**
4	***Cephalophus spadix***	**59**	**51**	**50**	**29**	**50**	**51**	**50**	**72**
5	***Cercocebus sanjei***	**71**	**95**	**113**	**142**	**124**	**142**	**136**	**130**
6	*Cercopithecus mitis*	21	9	12	21	18	26	27	41
7	*Civettictis civetta*	1	0	0	0	0	0	1	0
8	*Colobus angolensis*	1	2	1	2	3	1	3	1
9	***Cricetomys gambianus***	**215**	**293**	**301**	**267**	**334**	**405**	**328**	**333**
10	*Crocuta crocuta*	0	3	2	0	0	0	0	0
11	*Dendrohyrax validus*	23	39	34	48	51	31	48	55
12	***Genetta servalina***	**18**	**60**	**51**	**57**	**36**	**64**	**41**	**52**
13	*Hystrix africaeaustralis*	10	1	0	2	0	2	4	0
14	*Leptailurus serval*	0	0	1	0	0	0	0	0
15	*Loxodonta africana*	10	5	7	12	9	5	7	6
16	*Mellivora capensis*	6	6	7	13	11	9	16	24
17	*Mungos mungo*	2	7	1	0	2	9	2	1
18	*Nandinia binotata*	2	7	9	11	9	6	12	20
19	***Nesotragus moschatus***	**97**	**113**	**79**	**77**	**136**	**155**	**89**	**123**
20	*Orycteropus afer*	0	0	0	0	0	0	0	2
21	*Panthera pardus*	8	2	7	6	3	4	2	3
22	*Papio cynocephalus*	3	0	2	0	1	3	1	3
23	*Paraxerus vexillarius*	46	57	55	38	55	69	38	73
24	*Petrodromus tetradactylus*	2	6	29	11	10	1	16	4
25	*Potamochoerus larvatus*	18	23	16	21	24	42	23	30
26	*Procolobus gordonorum*	5	2	8	2	3	2	10	10
27	*Rhynchocyon cirnei*	4	3	8	0	1	6	5	4
28	***Rhynchocyon udzungwensis***	**45**	**74**	**81**	**57**	**65**	**50**	**53**	**46**
29	*Syncerus caffer*	4	4	3	3	7	1	4	0
30	*Thryonomys swinderianus*	0	0	2	4	1	1	0	0
31	*Tragelaphus scriptus*	0	4	2	7	2	3	7	7

## APPENDIX 2

Posterior parameter estimates (mean ± *SD*, and 95% Bayesian credible interval quantiles, BCI) for the *α* and *β* coefficients (logit scale) from the single‐species occupancy models of forest mammals in Mwanihana Forest, Udzungwa Mountains of Tanzania. Asterisks indicate no overlap of the 95% BCIs with zero

**Table:**

Species	Parameter	Mean (±*SD*)	95% BCI
Harvey's duiker	α0	0.896 (±0.377)	0.184 to 1.658
	α1	1.090 (±0.334)	0.429 to 1.730
	α_S_	−0.485 (±0.140)	−0.762 to −0.212*
	α_E_	−0.301 (±0.139)	−0.581 to −0.037*
	α_E2_	−0.235 (±0.130)	−0.485 to 0.027
	α_B_	0.246 (±0.303)	−0.358 to 0.828
	α2	2.067 (±0.420)	1.297 to 2.941
	*β*0	−0.140 (±0.041)	−0.221 to −0.061
	*β*_B_	0.138 (±0.041)	0.059 to 0.218*
Gray‐faced sengi	*α*0	−2.385 (±0.415)	−3.253 to −1.618
	*α*1	4.031 (±0.461)	3.175 to 4.984
	*α*_E_	0.998 (±0.213)	0.604 to 1.438*
	*α*_E2_	−0.184 (±0.195)	−0.580 to 0.190
	*α*_B_	−0.409 (±0.427)	−1.261 to 0.418
	*α*2	−0.982 (±0.362)	−1.692 to −0.272
	*β*0	−0.686 (±0.085)	−0.853 to −0.522
	*β*_B_	0.276 (±0.100)	0.082 to 0.470*
Suni	*α*0	−1.436 (±0.323)	−2.093 to −0.828
	*α*1	2.591 (±0.325)	1.982 to 3.251
	*α*_S_	0.013 (±0.122)	−0.229 to 0.256
	*α*_E_	−0.766 (±0.153)	−1.069 to −0.472*
	*α*_E2_	−0.372 (±0.142)	−0.656 to −0.100*
	*α*_B_	0.485 (±0.303)	−0.094 to 1.086*
	*α*2	0.148 (±0.312)	−0.462 to 0.763
	*β*0	−0.486 (±0.063)	−0.610 to −0.365
	*β*_B_	−0.122 (±0.066)	−0.251 to 0.004
Sanje mangabey	*α*0	1.114 (±0.378)	0.405 to 1.894
	*α*1	1.422 (±0.421)	0.641 to 2.297
	*α*_S_	0.263 (±0.144)	−0.011 to 0.550
	*α*_E_	0.135 (±0.130)	−0.116 to 0.392
	*α*_E2_	−0.535 (±0.134)	−0.802 to −0.273
	*α*_B_	−0.170 (±0.361)	−0.919 to 0.508
	*α*2	0.705 (±0.326)	0.084 to 1.362
	*β*0	−0.777 (±0.051)	−0.878 to −0.678
	*β*_B_	−0.022 (±0.051)	−0.120 to 0.079
Giant‐pouched rat	*α*0	0.347 (±0.288)	−0.208 to 0.926
	*α*1	1.950 (±0.266)	1.443 to 2.476
	*α*_S_	0.045 (±0.117)	−0.181 to 0.273
	*α*_E_	0.096 (±0.125)	−0.145 to 0.342
	*α*_E2_	0.126 (±0.119)	−0.104 to 0.362
	*α*_B_	−0.892 (±0.302)	−1.504 to −0.318*
	*α*2	0.008 (±0.283)	−0.548 to 0.563
	*β*0	0.199 (±0.043)	0.115 to 0.285
	*β*_B_	0.172 (±0.044)	0.086 to 0.259*
Bushy‐tailed mongoose	*α*0	2.120 (±0.545)	1.146 to 3.282
	*α*1	0.846 (±0.454)	−0.067 to 1.691
	*α*_S_	0.137 (±0.155)	−0.165 to 0.444
	*α*_E_	−0.452 (±0.163)	−0.778 to −0.139*
	*α*_E2_	−0.131 (±0.150)	−0.418 to 0.173
	*α*_B_	−0.721 (±0.434)	−1.635 to 0.069
	*α*2	1.209 (±0.342)	0.560 to 1.907
	*β*0	−0.095 (±0.040)	−0.173 to −0.017
	*β*_B_	−0.215 (±0.040)	−0.291 to −0.135*
Abbott's duiker	*α*0	0.405 (±0.574)	−0.603 to 1.676
	*α*1	0.856 (±0.702)	−0.522 to 2.275
	*α*_S_	0.032 (±0.184)	−0.329 to 0.395
	*α*_E_	0.819 (±0.245)	0.400 to 1.357*
	*α*_E2_	−0.226 (±0.171)	−0.566 to 0.104
	*α*_B_	−0.222 (±0.416)	−1.084 to 0.563
	*α*2	1.038 (±0.615)	0.025 to 2.462
	*β*0	−1.728 (±0.108)	−1.940 to −1.522
	*β*_B_	0.440 (±0.101)	0.245 to 0.637*
Lowe's genet	*α*0	−0.249 (±0.303)	−0.835 to 0.363
	*α*1	1.339 (±0.380)	0.635 to 2.119
	*α*_S_	0.144 (±0.129)	−0.106 to 0.404
	*α*_E_	0.003 (±0.155)	−0.314 to 0.298
	*α*_E2_	0.062 (±0.126)	−0.180 to 0.315
	*α*_B_	−0.493 (±0.304)	−1.114 to 0.079
	*α*2	−0.678 (±0.366)	−1.402 to 0.042
	*β*0	−1.493 (±0.095)	−1.680 to −1.312
	*β*_B_	0.338 (±0.111)	0.119 to 0.556*

Note

(*α*0 + *α*1) = intercept of occupancy; *α*_S_ = effect of the number of snares on occupancy; *α*_E_ = effect of elevation on occupancy; *α*_E2_ = effect of the square term of elevation on occupancy; *α*_B_ = effect of the firewood collection ban on occupancy; *α*2 = intercept for occupancy in the first year; *β*0 = intercept of detection probability; *β*_B_ = effect of the distance from park border on detection probability.

## APPENDIX 3

Significant effects of elevation on occupancy for the species camera‐trapped in Mwanihana Forest, Tanzania. Drawings by J. Kingdon reproduced with permission

## APPENDIX 4

Estimated mean occupancy and naïve occupancy for each analyzed species in Mwanihana Forest, Udzungwa Mountains National Park, in 2009–2016

**Table:**

Species	Estimated mean occupancy (Naïve occupancy)							
	2009	2010	2011	2012	2013	2014	2015	2016
Harvey's duiker	0.84 (0.80)	0.80 (0.73)	0.78 (0.76)	0.83 (0.73)	0.82 (0.72)	0.82 (0.82)	0.83 (0.83)	0.84 (0.83)
Gray‐faced sengi	0.29 (0.23)	0.30 (0.30)	0.33 (0.25)	0.29 (0.28)	0.29 (0.28)	0.29 (0.28)	0.30 (0.27)	0.28 (0.23)
Sanje mangabey	0.54 (0.50)	0.75 (0.67)	0.79 (0.68)	0.79 (0.71)	0.78 (0.68)	0.77 (0.75)	0.79 (0.70)	0.78 (0.70)
Suni	0.46 (0.42)	0.41 (0.38)	0.39 (0.35)	0.44 (0.37)	0.46 (0.46)	0.51 (0.48)	0.52 (0.42)	0.49 (0.47)
Bushy‐tailed mongoose	0.73 (0.70)	0.92 (0.92)	0.93 (0.85)	0.87 (0.85)	0.87 (0.80)	0.86 (0.86)	0.87 (0.83)	0.86 (0.82)
Abbott's duiker	0.66 (0.45)	0.65 (0.43)	0.65 (0.43)	0.61 (0.34)	0.60 (0.42)	0.60 (0.42)	0.60 (0.33)	0.60 (0.53)
Lowe's genet	0.36 (0.23)	0.56 (0.41)	0.62 (0.42)	0.53 (0.37)	0.50 (0.33)	0.50 (0.40)	0.50 (0.33)	0.49 (0.40)
Giant‐pouched rat	0.53 (0.52)	0.77 (0.77)	0.85 (0.77)	0.75 (0.68)	0.70 (0.67)	0.70 (0.70)	0.72 (0.65)	0.68 (0.68)
