# Supplementary material for: When parks work: Effect of anthropogenic disturbance on occupancy of tropical forest mammals
Source: Ecol Evol. 2020 Apr 16;10(9):3881–94. doi: 10.1002/ece3.6048 (PMC7244893; doi:10.1002/ece3.6048)
Supplement: Supplementary file 1 [file ECE3-10-3881-s001.pdf]

When parks work: effect of anthropogenic disturbance on occupancy of tropical forest mammals

Ecology and Evolution

Authors: Valentina Oberosler, Simone Tenan, Elise F. Zipkin, Francesco Rovero

Corresponding author: Valentina Oberosler

Tropical Biodiversity Section, MUSE - Museo delle Scienze, Corso del Lavoro e della Scienza 3, 38122 Trento, Italy

valentina.oberosler@muse.it

0039 0461 270374

Online resource S1. R and JAGS code for the single-species occupancy model fitted to the detection/non-detection data of eight mammal species. As an example, we provide the script for the Harvey's duiker (below).

load data

y_harvey: 3D matrix with the detection history for the Harvey's duiker, for each site i in sampling occasion j during year k

snares_std: standardized number of snares collected in each site i (array) during year k (year-site covariate)

elev: standardized elevation (site covariate)

elev2: standardized square term of elevation (site covariate)

border: standardized distance from park border (site covariate)

ban_35: binary covariate for the firewood collection activity status in each year k (year covariate)

```
modelFilename = "Harvey.txt"
cat("
   model {

   ## specify priors
   # Intercept psi_y1
   alpha6 <- log(mean.psi_y1/(1-mean.psi_y1))
   mean.psi_y1 ~ dunif(0,1)

   # Intercept psi_yy
   alpha0 <- log(mean.psi_yy/(1-mean.psi_yy))
   mean.psi_yy ~ dunif(0,1)

   # Intercept p
   beta0 <- log(mean.p/(1-mean.p))
   mean.p ~ dunif(0,1)

   # occupancy
   alpha1 ~ dnorm(0,0.01)
   alpha2 ~ dnorm(0,0.01)
   alpha3 ~ dnorm(0,0.01)
   alpha4 ~ dnorm(0,0.01)
   alpha5 ~ dnorm(0,0.01)

   # detection
   beta1 ~ dnorm(0,0.01)

   ##ecological submodel
   for(i in 1:nsite){

   z[i,1] ~ dbern(psi[i,1])

   logit(psi[i,1]) <- alpha6 + alpha2 * snares_std[i,1] + alpha3 * elev[i] + alpha4 * elev2[i]

   for(k in 2:nyear){
   z[i,k] ~ dbern(psi[i,k])
   }  }
```

```
for(i in 1:nsite){
for(k in 2:(nyear)){
logit(psi[i,k]) <- alpha0 + alpha1 * z[i,k-1] + alpha2 *snares_std[i,k] + alpha3 * elev[i] + alpha4 * elev2[i]
                + alpha5 * ban_35[k]
} }

# observational model
for(i in 1:nsite){
for(j in 1:nrep){
for(k in 1:nyear){

muy[i,j,k] <- z[i,k]*p[i]

y[i,j,k] ~ dbern(muy[i,j,k])
} } }

for(i in 1:nsite){
logit(p[i]) <- beta0 + beta1 * border[i]  }

## derived parameters
n.occ[1]<- sum(z[1:nsite,1])
p_mean<-mean(p[])

for(k in 2:nyear){
n.occ[k] <- sum(z[1:nsite,k])
}

for(k in 1:nyear){
psi_year[k]<-mean(psi[,k])
} }
## end model
", fill=TRUE, file=modelFilename)

# data for JAGS
bugs.data <- list(y=y, nsite=dim(y)[1], nrep=dim(y)[2], nyear=dim(y)[3], elev=elev, elev2=elev2,
border=border, snares_std=snares_std, ban_35=ban_35)
```

```
## parameters to monitor
params <-c("psi_year","n.occ","p_mean","alpha0","alpha1","alpha2","alpha3","alpha4","alpha5",
"alpha6","beta0","beta1","mean.psi_y1","mean.psi_yy","mean.p")

## inits
zst <- apply(y_harvey, c(1, 3), sum, na.rm=T)
zsti<-ifelse(zst>0,1,0)

jags.inits <- function(){ list(z = zsti,
                    mean.psi_y1=runif(1,0,1), mean.psi_yy=runif(1,0,1), mean.p=runif(1,0,1),
                    alpha1 = runif(1, -3, 3), alpha2 = runif(1, -3, 3), alpha3 = runif(1, -3, 3),
                    alpha4 = runif(1, -3, 3), alpha5 = runif(1, -3, 3), beta1 = runif(1, -3, 3) )}

# MCMC settings
n.adapt <- 1000
n.burnin <- 5000
n.iter <- 100000
n.thin <- 20
n.chains <- 3
start.time = Sys.time()

## adaptive phase to maximise MCMC efficiency
out_harvey_final <- jags(data = bugs.data,
                    inits=jags.inits,
                    params,
                    "Harvey.txt",
                    n.chains=n.chains,
                    n.adapt=n.adapt,
                    n.thin=n.thin,
                    n.iter=n.iter,
                    n.burnin=n.burnin)
end.time = Sys.time()
elapsed.time = round(difftime(end.time, start.time, units='mins'),2)
cat(paste(paste('Posterior computed in ', elapsed.time, sep=' '),'minutes\n', sep=' '))

#### END
```
